# Clinical Features of Craniopharyngioma With Tumoral Hemorrhage: A Retrospective Case-Controlled Study

**DOI:** 10.3389/fsurg.2022.845273

**Published:** 2022-03-14

**Authors:** Yusi Chen, Feng Hu, Junwen Wang, Kuan Huang, Weihua Liu, Yutang Tan, Kai Zhao, Qungen Xiao, Ting Lei, Kai Shu

**Affiliations:** Department of Neurosurgery, Tongji Hospital, Tongji Medical College, Huazhong University of Science and Technology, Wuhan, China

**Keywords:** craniopharyngioma, endocrine, pituitary hormone, risk factor, tumoral hemorrhage

## Abstract

**Background:**

Craniopharyngioma (CP) with tumoral hemorrhage is a very rare syndrome presenting with various manifestation and unfavorable outcomes. The current retrospective study was performed to summarize the clinical features of CP with tumoral hemorrhage.

**Methods:**

In this study, 185 patients with pathological diagnosis of CP (18 patients with hemorrhage) were enrolled. Clinical characteristics, radiological and surgical treatments, and post-operative complications were analyzed. In addition, the correlations between sexual hormones and tumor volume were explored.

**Results:**

Drowsiness, acute syndrome, and pituitary deficiency were more frequent in patients with hemorrhage patients. Prothrombin time (PT) were higher in patients with hemorrhage. Luteinizing hormone (LH) and testosterone (T) were lower in male patients with hemorrhage. Post-operative electrolyte disturbances, hypothalamic syndrome, and death appeared more frequently in the hemorrhage group. Moreover, prolactin (PRL) and cortisol 8AM were found to be correlated with the volume of the tumor and the hematoma, respectively.

**Conclusion:**

The current study presented the clinical features of CP apoplexy from the aspects of clinical characteristics, radiography, surgical treatment, and post-operative complications. Patients with CP apoplexy could benefit from the proper processing of peritumoral hemorrhage and post-operative monitoring of the electrolyte.

## Introduction

Craniopharyngiomas (CPs) are primary central nervous system tumors originating from the remnant of epithelial tissue of the craniopharyngeal duct in the embryonic period (1). Most CPs are considered benign but highly recurrent, which locate at the sella turcica and suprasellar region with about half of them originating at the third ventricle floor (2). The incidence of CP has a bimodal age distribution with peaks at 5~14 and 50~74 years old (3). Critical structures, such as the pituitary gland, hypothalamus, and chiasm are often involved during the development of CPs, which gives rise to the symptoms of endocrine disorders, diabetes insipidus (DI), and visual disorders.

Craniopharyngioma tumoral hemorrhage is a very rare syndrome induced by a hemorrhagic lesion from the CP. The clinical manifestation of acute hemorrhage in CP, especially for intrasellar CP, resembles that of pituitary tumor apoplexy, such as the acute onset of visual disorders and pituitary or hypothalamic deficiency. The mass effect caused by the subdural hematoma or the edematous parenchyma in CP hemorrhage exerts further damage to neighboring structures. When the hematoma blocks up the intraventricular foramen or enters the third ventricle with protrusion of the tumor, expansion of the ventricular system and hydrocephalus can appear. For pituitary adenomas apoplexy, hypertension, acute increase of intracranial pressure (ICP), angiographic procedures, and dynamic tests have been identified as risk factors (4, 5). However, detailed clinical features of CP hemorrhage have remained unclear so far.

By comparing CP patients with and without tumoral hemorrhage, the current study was designed to illustrate a detailed overview of the clinical features of CP tumoral hemorrhage from the data of patients with CP retrospectively, such as pre-operative laboratory tests, radiological results, surgical treatment, and course records.

## Methods

Clinical data of 185 patients receiving resective operation with a pathological diagnosis of CP in Affiliated Tongji Hospital of Tongji Medical College, Huazhong University of Science and Technology, Wuhan, China between January 2013 and February 2021 were collected. Among these patients, 18 of them were recognized as CP with tumoral hemorrhage with radiographical and pathological diagnosis. Our protocol was designed as a single-centered retrospective study, and we have thoroughly followed the principles of medical research involving human subjects expressed in the Declaration of Helsinki. Informed consent of the patients has been obtained after a full explanation of the purpose and the nature of all procedures used. Ethical approval was received from the institutional review board of Tongji Hospital (TJ-IRB20210523). This work has been reported according to the STROCSS criteria (6).

Patients with these situations were excluded from analysis: (1) patients who had a non-CP or unverified pathological diagnosis at discharge. (2) Patients who received surgical or radio-oncological treatment on the sellar region before current admission. (3) Patients who received treatment of coagulation modulation, immunosuppressive agents, or chemotherapeutic drugs. (4) Patients who had a CP combined with other clinical emergencies, such as acute myocardial infarction, cerebral stroke, trauma, and sepsis at admission. (5) Patients who were diagnosed with other tumors, hematological diseases, autoimmune diseases, liver failure, or renal failure.

For each case, age, gender, smoking status, alcohol intake, past history, symptoms, blood pressure, blood cell count, blood biochemical tests, coagulation tests, hormone tests, radiological results, histologic types, surgical treatment, and the outcome at discharge were collected. The first test result after admission and before the operation was taken for the items of blood tests in the analysis.

The images of CT and MRI were collected. The degree of hypothalamic involvement was evaluated on the sagittal view of pre-operative CT according to the classification standard applied in the study of Puget et al. (7). Grade 0 refers to no hypothalamic involvement. Grade 1 refers to a tumor abutting or displacing the hypothalamus. Grade 2 refers to hypothalamic involvement. Regions of interest (ROI) on each slice of the DICOM image were circumscribed by software ImageJ 1.51. The area of ROIs and corresponding thickness of slices were collected for volume calculation of the tumor and the hematoma. Results of the area of ROI times the thickness for each slice were summed up as the total volume, which was performed according to the protocol of a previous research (8).

In the analysis of clinical characteristics, categorical, ordinal, and continuous variables were compared between groups with chi-squared test, Mann–Whitney *U*-test, and Student's *t*-test, respectively. Continuous variables were tested for normalized distribution with the chi-squared test before intergroup comparison. Scatter plot and linear regression were performed for the correlation between volume variables (tumor and hematoma) and seven hormones [luteinizing hormone (LH), follicle stimulating hormone (FSH), prolactin (PRL), estradiol (E_2_), progesterone (P), testosterone (T), and cortisol 8AM]. A value of *p* < 0.05 was recognized as statistically significant. All statistical analyses were performed using SPSS Version 22.0. Figures were plotted with GraphPad Prism 7.

## Results

### Clinical Characteristics

We included 185 CP patients in current study, with 42 (26.58%) patients aged 0~18 years and 143 (73.42%) patients aged >18 years. It was found that 20 patients were of pubertal status (aged 8~13 years for women and 9~14 years for men). Of the 185 patients, 112 were men (60.54%) and 73 were women (39.46%). Patients were divided into non-hemorrhage group (*N* = 167, 90.27%) and hemorrhage group (*N* = 18, 9.73%) (Table 1). No significant differences were found in the items of age, gender, and past history. For symptoms, visual disorders were more frequent in non-hemorrhagic patients at admission (*p* = 0.016). Within three types of visual disorders analyzed, visual acuity decline appeared as the most frequent visual symptom in both groups (59.28% in the non-hemorrhage group and 33.33% in the hemorrhage group) and as the only subtype with a significant intergroup difference (*p* = 0.035). Drowsiness appeared more in patients with CP hemorrhage (*p* = 0.002). No differences between the two groups were detected for other symptoms (DI, weight gain/loss, weakness of limbs, hypomnesia, nausea and vomiting, cranial neuropathies, and pituitary deficiency). Acute symptoms within 3 days appeared significantly more frequent in patients with hemorrhage (33.33%) than the counterpart (5.99%, *p* < 0.001). In the terms of blood pressure, blood cell count, and blood biochemical tests, non-hemorrhagic and hemorrhagic patients presented similar patterns. Patients with tumoral hemorrhage had a trend to present a lower level of red blood cell (RBC) count (*p* = 0.111) and a higher level of blood glucose (*p* = 0.114). Coagulation and hormone tests revealed that patients with tumoral hemorrhage had significantly longer prothrombin time (PT) (*p* = 0.006) and lower LH (*p* < 0.001) levels than the counterpart. Male patients suffering hemorrhage showed a lower LH level and lower T level in the blood serum (both *p* < 0.001) than non-hemorrhagic men. Abnormality in at least two items of pituitary hormone tests was able to distinguish patients with hemorrhage from the other group (*p* = 0.030).

**Table 1:** Baseline characteristics in patients with Craniopharyngioma (CP).

	**Non-hemorrhage group, *N* = 167**	**Hemorrhage group, *N* = 18**	***P***
Age	36.92 ± 18.52	36.50 ± 21.00	0.929
Gender (male), *n* (%)	100 (59.88)	12 (66.67)	0.576
Hypertension, *n* (%)	13 (7.78)	2 (11.11)	0.624
Diabetes Mellitus, *n* (%)	4 (2.40)	0 (0.00)	0.508
Headache, *n* (%)	88 (52.69)	8 (44.44)	0.473
Visual disorders, *n* (%)	104 (62.28)	6 (33.33)	**0.016**
Visual acuity decline, *n* (%)	99 (59.28)	6 (33.33)	**0.035**
Bitemporal hemianopia, *n* (%)	12 (7.19)	1 (5.56)	0.798
Diplopia, *n* (%)	6 (3.59)	0 (0.00)	0.415
Diabetes insipidus, *n* (%)	24 (14.37)	4 (22.22)	0.379
Weight gain/loss, *n* (%)	10 (5.99)	1 (5.56)	0.941
Drowsiness, *n* (%)	11 (6.59)	5 (27.78)	**0.002**
Weakness of limbs, *n* (%)	19 (11.38)	2 (11.11)	0.973
Memory impairment, *n* (%)	11 (6.59)	1 (5.56)	0.866
Nausea and vomiting, *n* (%)	39 (23.35)	6 (33.33)	0.350
Cranial neuropathies, *n* (%)	8 (4.79)	1 (5.56)	0.787
Growth retardation, *n* (%)	4 (2.40)	1 (5.56)	0.433
Abnormal menstruation^*^, *n* (%)	6 (8.96)	1 (16.67)	0.542
Sexual dysfunction, *n* (%)	5 (2.99)	0 (0.00)	0.458
Acute symptoms within 3 days, *n* (%)	10 (5.99)	6 (33.33)	**<0.001**
SBP (mmHg)	117.51 ± 14.95	119.11 ± 15.01	0.666
DBP (mmHg)	76.13 ± 10.83	75.61 ± 11.00	0.847
RBC count (10^9^/L)	4.43 ± 0.63	4.24 ± 0.50	0.111
WBC count (10^9^/L)	6.76 ± 2.77	6.98 ± 2.47	0.742
PLT count (10^9^/L)	231.62 ± 76.59	213.56 ± 53.31	0.331
Hemoglobin (mg/dL)	131.53 ± 15.40	125.89 ± 12.84	0.136
TC (mmol/L)	4.65 ± 1.20	4.51 ± 0.95	0.621
TG (mmol/L)	1.57 ± 1.25	1.46 ± 1.00	0.700
Blood glucose (mmol/L)	5.16 ± 1.37	5.71 ± 1.66	0.114
PT (s)	13.17 ± 0.81	13.73 ± 0.89	**0.006**
APTT (s)	37.62 ± 5.39	39.86 ± 8.03	0.115
Fibrinogen (g/L)	3.13 ± 0.74	3.39 ± 0.58	0.157
LH (IU/L)	3.00 ± 4.40	0.90 ± 0.82	**<0.001**
Male	2.34 ± 2.29	0.71 ± 0.48	**<0.001**
Female	3.99 ± 6.26	1.26 ± 1.24	0.294
FSH (IU/L)	6.92 ± 11.96	3.78 ± 4.78	0.271
Male	3.68 ± 3.80	2.42 ± 1.79	0.260
Female	11.75 ± 17.28	6.49 ± 7.58	0.464
PRL (μg/L)	22.17 ± 17.12	18.54 ± 10.33	0.379
Male	17.49 ± 11.31	18.52 ± 10.04	0.765
Female	29.15 ± 21.50	18.58 ± 11.88	0.240
Female E_2_ (pg/mL)	44.39 ± 64.44	27.54 ± 9.80	0.527
Female P (μg/L)	0.85 ± 1.92	0.22 ± 0.20	0.430
Male T (μg/L)	1.54 ± 2.02	0.23 ± 0.23	**<0.001**
Cortisol 8AM (μg/dL)	8.32 ± 6.55	7.08 ± 5.30	0.439
TSH (mIU/L)	1.84 ± 1.39	1.79 ± 1.81	0.888
Pituitary deficiency^**^, *n* (%)	95 (56.89)	15 (83.33)	**0.030**

*APTT, activated partial thromboplastin time; CN, cranial nerve; DBP, diastolic blood pressure; E_2_, estradiol; FSH, follicle stimulating hormone; FT_3_, free triiodothyronine; FT_4_, free thyroxine; LH, luteinizing hormone; P, progesterone; PLT, platelet; PRL, prolactin; PT, prothrombin time; RBC, red blood cell; SBP, systolic blood pressure; T, testosterone; TC, total cholesterol; TG, triglyceride; TSH, thyroid stimulating hormone; WBC, white blood cell*.

*Data are presented as mean ± SD or n (%). Bolded p-values show statistical significances with p < 0.05*.

* *Percentage and the value of p adjusted for female patients*.

** *Cases with abnormality in at least two items of pituitary hormone tests were regarded as pituitary deficiency*.

The surgical, radiological, and histologic characteristics of patients with CP were collected in Table 2. Of the 185 patients, 160 patients (86.49%) were treated with craniotomy and 161 patients (87.03%) realized gross total resection (GTR). The operative approach, GTR rate, and tumor-involved regions did not distinguish patients with hemorrhage from non-hemorrhage ones. The distribution of the severity of hypothalamic involvement was similar in the two groups. The largest part of the patients, no matter with or without hemorrhage, showed displacement of the hypothalamus caused by the tumor. Chiasm compression was more presented in patients with tumoral hemorrhage (*p* = 0.024). For histologic types, the group with hemorrhage contained 83.33% adamantinomatous and 16.67% patients with papillary CP. The group without hemorrhage contained 88.62% adamantinomatous and 11.38% patients with papillary CP. No significant differences were found between the two groups. Furthermore, we collected the data of other surgical treatments besides tumor resection showing that burr hole drainage, as well as post-operative hematoma evacuation, were more applied in the hemorrhage group (*p* = 0.027 and *p* = 0.006, respectively). Post-operative complications before the discharge were also reported. It was noted that patients with hemorrhage had higher incidence rates of electrolyte disturbances (*p* = 0.002), hypothalamic syndrome (*p* = 0.021), and death (*p* = 0.046). No significant differences were discovered in the terms of DI, pituitary deficiency, and visual disorders.

**Table 2:** Surgery, radiographic and histologic characteristics, and post-operative complications in CP patients.

	**Non-hemorrhage group, *N* = 167**	**Hemorrhage group, *N* = 18**	***P***
Operation type			0.274
Craniotomy, *n* (%)	146 (87.43)	14 (77.78)	
Transsphenoidal, *n* (%)	21 (12.57)	4 (22.22)	
Gross total resection			
Craniotomy, *n* (%)	128 (87.67)	12 (85.71)	0.688
Transsphenoidal, *n* (%)	18 (85.71)	3 (75.00)	0.600
Tumor-involved regions			
Sella turcica, *n* (%)	107 (64.07)	13 (72.22)	0.491
Suprasellar, *n* (%)	107 (64.07)	11 (61.11)	0.804
Parasellar, *n* (%)	4 (2.40)	1 (5.56)	0.404
Third ventricle, *n* (%)	18 (10.78)	1 (5.56)	0.489
Hypothalamic involvement			0.915
Grade 0	21 (12.57)	2 (11.11)	
Grade 1	82 (49.10)	9 (50.00)	
Grade 2	64 (38.32)	7 (38.89)	
Ventricle system enlargement, *n* (%)	28 (16.77)	3 (16.67)	0.991
Chiasm compression, *n* (%)	57 (34.13)	11 (61.11)	**0.024**
Histologic types			0.529
Adamantinomatous CP	148 (88.62)	15 (83.33)	
Papillary CP	19 (11.38)	3 (16.67)	
Burr hole drainage, *n* (%)	7 (4.19)	3 (16.67)	**0.027**
Post-operative hematoma evacuation, *n* (%)	2 (1.20)	2 (11.11)	**0.006**
V-P shunt, *n* (%)	2 (1.20)	0 (0.00)	0.642
Tracheotomy, *n* (%)	8 (4.79)	1 (5.56)	0.886
Post-operative complications			
Diabetes insipidus			
Transient, *n* (%)	29 (17.37)	1 (5.56)	0.198
Permanent, *n* (%)	8 (4.79)	2 (11.11)	0.261
Pituitary deficiency, *n* (%)	18 (10.78)	2 (11.11)	0.966
Electrolyte disturbances, *n* (%)	15 (8.98)	6 (33.33)	**0.002**
Hypothalamic syndrome, *n* (%)	3 (1.80)	2 (11.11)	**0.021**
Visual disorders, *n* (%)	28 (16.77)	1 (5.56)	0.215
Death, *n* (%)	1 (0.60)	1 (5.56)	**0.046**

*V-P shunt, ventriculo-peritoneal shunt*.

*Data are presented as mean ± SEM or n (%). Bolded p show statistical significances with p < 0.05*.

**Figure 1:**
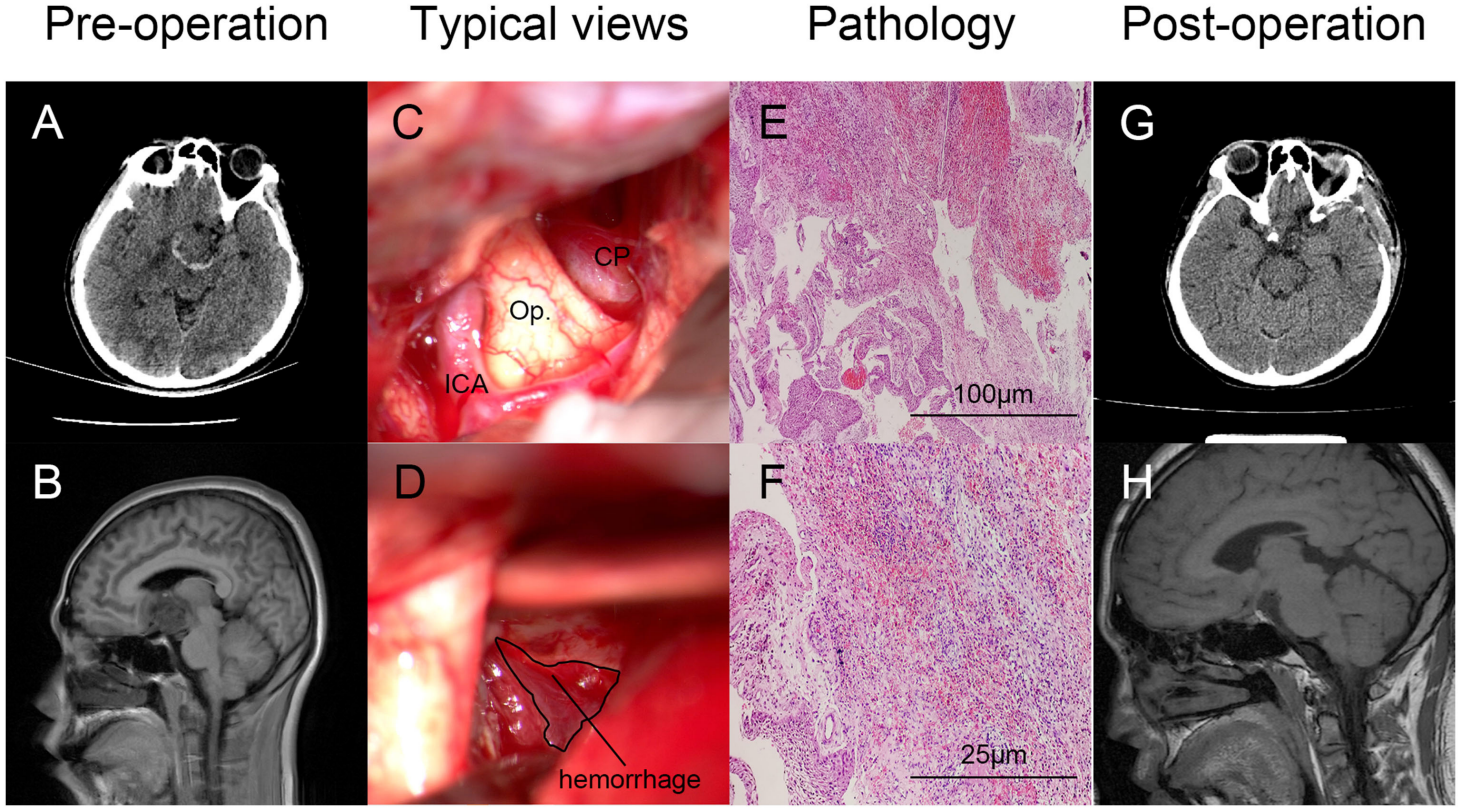
Typical case illustration of craniopharyngioma (CP) with tumoral hemorrhage. Pre-**(A,B)** and post-operative **(G,H)** radiological images (axial CT and sagittal MRI) showed the hemorrhage around the lesion **(A)** and gross total resection (GTR) of the tumor. Intraoperative views of the tumor and hemorrhage around the tumor of Patient No. 2 were displayed as a typical case **(C,D)**. Hemorrhage was labeled with a circle. Pathological diagnosis confirmed the tumor as suprasellar CP with tumoral hemorrhage [**(E)**, HE, ×100; **(F)** HE, ×400). CP, craniopharyngioma; ICA, internal carotid artery; Op., optic nerve.

Clinical characteristics, such as age, gender, main symptoms, past history, hormone abnormality, tumor-involved regions, surgical treatments, and outcome at the discharge of 18 CP patients with tumoral hemorrhage are presented in Table 3. Pre- and post-operative radiological images, typical views of the tumoral hemorrhage, and pathological results of Patient No. 2 are displayed as a typical case in Figure 1.

**Table 3:** Summary of 18 cases of CP with intratumoral hemorrhage.

**Patient No**.	**Age (years)**	**Gender**	**Main symptoms**	**Past history**	**Hormone abnormality**	**Tumor-involved regions**	**Surgical treatment**	**Outcome**
1	26–30^*^	M	Headache, visual acuity decline in 20 days, drowsiness	Smoking	LH↓	Sella turcica	Craniotomy (GTR)	Improvement
2	21–25	M	Visual acuity decline, nausea, and vomiting, DI, drowsiness, progressive weakness of limbs in 3 days, weight gain		LH↓, FSH↓, T↓	Suprasellar	Craniotomy (GTR)	DI (temp), pituitary insufficiency (temp)
3	6–10	F	Headache, growth retardation, shaking hands for 2 weeks		N/A	Third ventricle	Craniotomy (GTR)	Death
4	6–10	M	Fever, convulsion in 3 days, altered consciousness		LH↓, FSH↓, T↓	Sella turcica	Craniotomy, hematoma evacuation, tracheotomy	Hypothalamic insufficiency
5	51–55	M	Headache for 3 years with deterioration in 1 year, DI		LH↓, PRL↑, T↓	Sella turcica	Craniotomy (GTR)	DI (temp), electrolyte disturbances (temp)
6	36–40	M	Headache, visual acuity decline in 3 months, DI		Cortisol↓	Sella turcica, suprasellar	Transsphenoidal (GTR)	Improvement
7	31–35	F	Progressive headache in 2 weeks, amenorrhea		LH↓, FSH↓, P↓, Cortisol↓	Sella turcica, suprasellar	Craniotomy (GTR)	Improvement
8	61–65	M	Severe headache with visual acuity decline in 10 days		LH↓, T↓, Cortisol↓	Sella turcica, suprasellar, chiasm	Craniotomy (GTR)	Electrolyte disturbances (temp)
9	56–60	F	Deteriorated dizziness in 1 week, vomiting, convulsion, altered consciousness	Hypertension	P↓	Sella turcica	Transsphenoidal (GTR)	Electrolyte disturbances (temp)
10	11–15	F	Headache, DI, deteriorated nausea and vomiting in 3 days, altered consciousness		LH↓, FSH↓	Suprasellar	Craniotomy, burr hole drainage	Pituitary insufficiency (temp), electrolyte disturbances (temp)
11	45–50	M	Headache for over 2 years, dizziness			Sella turcica	Transsphenoidal (GTR)	Improvement
12	61–65	F	Visual acuity decline in 20 days, bitemporal hemianopia	Hypertension, hyperlipemia		Suprasellar	Craniotomy	Vision decline, electrolyte disturbances (temp)
13	6–10	M	Headache in 1 week, nausea and vomiting		LH↓, FSH↓, T↓	Sella turcica, suprasellar	Craniotomy (GTR)	Improvement
14	31–35	M	Headache, nausea, and vomiting in 2 days	Smoking, alcohol intake	N/A	Parasellar	Craniotomy, hematoma evacuation	Epidural hematoma (temp)
15	66–70	M	Dizziness, drowsiness, tinnitus, hypomnesia for 1 year	Smoking, alcohol intake	LH↓	Sella turcica, suprasellar	Craniotomy (GTR)	Improvement
16	16–20	M	Altered consciousness in 22 h		T↓, Cortisol↓	Sella turcica, suprasellar	Craniotomy, burr hole drainage	Improvement
17	61–65	F	Headache deteriorated in 2 days, visual acuity decline, nausea, and vomiting		LH↓, P↓, Cortisol↓	Sella turcica, suprasellar	Transsphenoidal	Improvement
18	30–35	M	Weakness of limbs and progressive convulsion in 1 week		LH↓, FSH↓, T↓	Sella turcica, suprasellar	Craniotomy (GTR), burr hole drainage	Electrolyte disturbances (temp)

*DI, diabetes insipidus; FSH, follicle stimulating hormone; GTR, gross total resection; LH, luteinizing hormone; N/A, not available; P, progesterone; PRL, prolactin; T, testosterone; temp, temporary. ↓ and ↑ refers to a decreased level and an increased level of the hormones respectively*.

* *Age was amended to avoid the identification of a certain individual*.

### Correlation Between Volume Variables and Hormones

**Figure 2:**
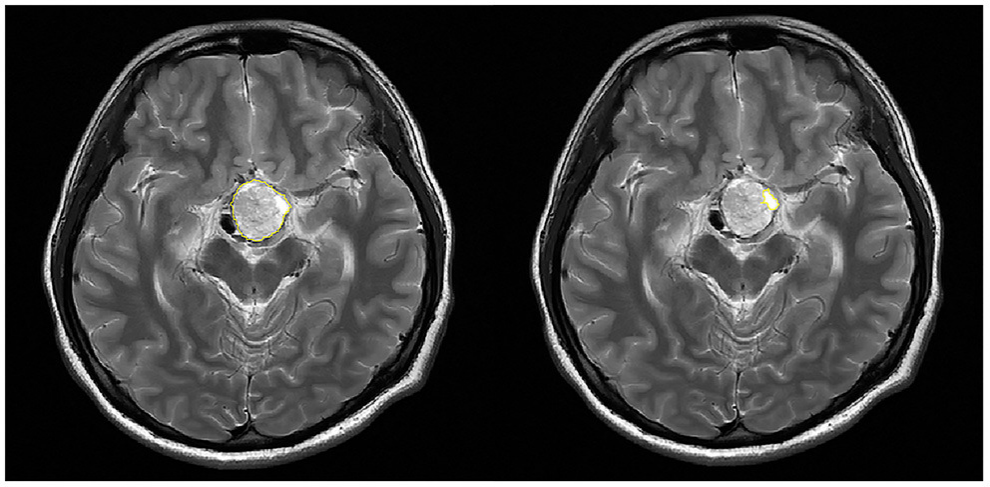
Selection of the region of interest (ROI) in the calculation of volumes of the tumor and the hematoma. The tumor (Left) or the hematoma (Right) area on each slice of the DICOM image was recognized and circumscribed (yellow circle) with the software.

**Figure 3:**
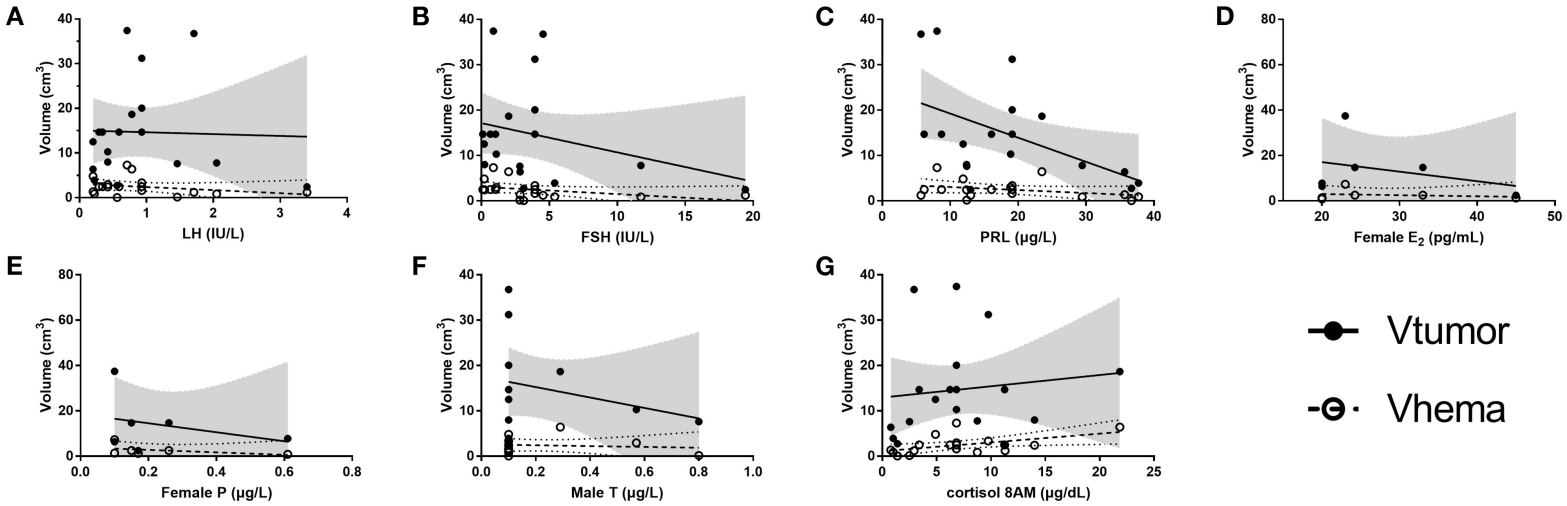
Correlation between the tumor/ hematoma volumes and the hormones. **(A)** LH, **(B)** FSH, **(C)** PRL, **(D)** female E_2_, **(E)** female P, **(F)** male T, and **(G)** Cortisol 8AM. E2, estradiol; FSH, follicle stimulating hormone; LH, luteinizing hormone; P, progesterone; PRL, prolactin; T, testosterone; Vhema, the volume of the hematoma; Vtumor, the volume of the tumor. The values of *r* and *p* of V tumor for hormones are: LH (*r* = −0.0313, *p* = 0.902), FSH (*r* = −0.2868, *p* = 0.249), PRL (*r* = −0.5114, *p* = 0.030), female E_2_ (*r* = −0.3306, *p* = 0.522), female P (*r* = −0.3091, *p* = 0.551), male T (*r* = −0.2579, *p* = 0.418), and cortisol 8AM (*r* = 0.1223, *p* = 0.629). The *r* values and *p* of V hema for hormones are: LH (*r* = −0.2848, *p* = 0.252), FSH (*r* = −0.3831, *p* = 0.117), PRL (*r* = −0.3439, *p* = 0.162), female E_2_ (*r* = −0.1999, *p* = 0.704), female P (*r* = −0.4386, *P* = 0.384), male T (*r* = −0.1151, *p* = 0.722), and cortisol 8AM (*r* = 0.5155, *p* = 0.029). Gray shadow and dotted curves represent the 95% confidence bands of the best-fit line for the volume of the tumor and the hematoma, respectively.

For 18 patients with hemorrhage, the serum levels of seven hormones (E_2_ and P for female only, and T for male only) were collected and volumes of the tumor and the hematoma were calculated. The selection of ROI is illustrated in Figure 2. As shown in Figure 3, scatter plots and linear regression were performed to evaluate the correlation between pre-operative hormone levels and volumes of the tumor and the hematoma. Among the hormones, lower PRL level was correlated with higher tumor volume (*r* = −0.5114, *p* = 0.030, Figure 3C), while the higher level of cortisol 8AM was correlated with higher hematoma volume (*r* = 0.5155, *p* = 0.029, Figure 3G). No significant correlations were discovered between other hormones and the volume variates.

## Discussion

The current study presented the clinical features of CP with tumoral hemorrhage. We displayed the detailed clinical features of patients with CP hemorrhage and compared them with non-hemorrhagic patients. Visual disorders, drowsiness, acute symptoms in 3 days, high PT, low LH, and pituitary deficiency significantly differentiate cases with hemorrhage from non-hemorrhage ones. Chiasm compression was more often in patients with hemorrhage. For post-operative complications, electrolyte disturbances, hypothalamic syndrome, and death had higher incidence rates in patients with hemorrhage. Moreover, a negative correlation between PRL and the tumor volume and a positive correlation between cortisol 8AM and the hematoma volume were found.

Tumoral hemorrhage in CP appears with diverse clinical manifestation and unfavorable outcomes. When compared with pituitary adenoma apoplexy, little is known or reported about the clinical features of CP tumoral hemorrhage. Here, we would like to define CP with tumoral hemorrhage as CP apoplexy. In the current study, our data showed that the incidence rate of CP apoplexy was 9.73%, similar to that of pituitary apoplexy (5). According to the summary of cases with hemorrhage, CP apoplexy also presented common features of hemorrhage in other intracranial tumors, such as the acute onset of headache, nausea/vomiting, fever, convulsion, and altered consciousness. Some patients developed the symptoms of pituitary deficiency and visual disorders (9–11). Our data revealed significant intergroup differences in acute symptoms within 3 days and pituitary deficiency.

It is also noteworthy that some patients with subclinical hemorrhage did not have acute onset but showed dizziness, drowsiness, weakness of limbs, shaking hands, and hypomnesia, which appeared in some of our patients and may be concerned with slowly increased ICP (12, 13). According to a previous study, increased ICP and hydrocephalus appeared significantly more often in CP patients with cerebral infarction and was recognized as a predictive factor of this severe complication (3). The rapid shift of ICP was focused in the prevention of cerebral infarction in patients with CP (14). Interestingly, hydrocephalus or ventricle system enlargement showed no differences between the hemorrhage group and the non-hemorrhage group in our study, which may be related to the steady elevation of ICP.

Notice that visual disorders showed a significant intergroup difference but appeared more frequently in the non-hemorrhage group. This result may come from the incooperation of vision examination in patients with consciousness disorders. We collected the data of four patients with consciousness disorders in the hemorrhage group and none in the non-hemorrhage group.

For biochemical tests, prolonged PT appeared more frequent in patients with hemorrhage. PT has been identified as a predictor for ischemic stroke, intracranial hemorrhage (ICH) (15–17). As an indicator of the extrinsic pathway of the coagulation cascade, prolonged PT means the consumption of pro-coagulant factors, which in CP apoplexy may result from the initiation of the coagulation system due to microvessel rupture or the tissue factor released from the tumor during necrosis.

For neuroradiological results and surgical treatments, chiasm compression was the only imaging feature that showed difference between the two groups. Patients with CP apoplexy had a higher incidence, which may result from the extra mass effect of the hematoma.

During the operation, we observed that hematoma wrapped around the tumor and presented a crescent or ring shape on CT and MRI in most of the patients with CP apoplexy. The feature of peritumoral hematoma is rare and different from intratumoral hemorrhage which is commonly found inside the capsule similar to the pituitary apoplexy. The hematoma may come from the small branches distributing on the tumor capsule from the anterior communicating artery complex that supplies the suprasellar part of the tumor (18), which is different from the pituitary adenoma that is supplied by the pituitary portal system inside the pituitary. This hematoma can stimulate the structure within the sellar region and induce acute symptoms, such as acute visual disturbance or hypothalamic symptom. Patients with pituitary macroadenomas and suprasellar invasion have a higher risk of subarachnoid hemorrhage when the capsule extends into the suprasellar subarachnoid space where blood supply is abundant (19). It was shown that 11 patients in our hemorrhage group presented suprasellar invasion with the featured presentation, so subarachnoid hemorrhage may be related to the peritumoral hematoma. Moreover, the mass effect of CP can further lead to the compression on peritumoral edematous parenchymal and cause intraparenchymal hemorrhage that also contributes to the hematoma. Therefore, attention should be paid to the aspiration of blood or clots surrounding the tumor when accessing and separating the tumor capsule.

The general aim of CP surgery is “maximal but hypothalamic-sparing resection” (20). CP is mainly treated surgically by craniotomy or transsphenoidal endoscopic surgery. Based on recent studies, there is no significant difference in the extent of resection, recurrence, and the necessity for adjuvant therapy (21). Cerebrospinal fluid (CSF) leakage has been thought to be higher in the transsphenoidal group, while a previous study of transsphenoidal approach reported an incidence rate of 14.6% (22). In our center, we apply basically transsphenoidal microsurgery for intrasellar CPs while transcranial approach for other CPs. The selection of approaches showed no significant influences on the GTR rate in both hemorrhage and non-hemorrhage groups. Since the number of patients applied with transsphenoidal approach was small, it was difficult to make a conclusion from the comparison of GTR rate of different approaches. The transsphenoidal approach might be equally suitable approach to large sellar and suprasellar pathologies, such as hemorrhagic ones reported in the current study and pituitary apoplexy in a previous study (23). Similar proportion of endocrine complications was observed in transsphenoidal and transcranial approaches (24). GTR or subtotal resection were selected according to the tumor involvement and the endocrinal function. Recurrence was more observed in studies with lower GTR (24).

The histologic subtypes of CP have been reported to affect the tumor solidness and the strength of angiogenesis, which may also affect the incidence rate of tumoral hemorrhage (25, 26). However, differences in the histologic distribution were not found between the hemorrhage group and the non-hemorrhage group. This phenomenon may underline the explanation that ruptures of small vessels on the capsule cause the hematoma as the tumor grows.

Patients with tumoral hemorrhage had a higher incidence rate of post-operative electrolyte disturbances, hypothalamic syndrome, and death. Post-operative electrolyte disturbances had been recognized as a risk factor of cerebral infarction in CP (14). This may represent a state of pituitary or hypothalamic disorder and necessitate continuous monitoring as well as individualized electrolyte supplementation. Apart from CP apoplexy, the origin location or the extend of compression on the infundibulum can also affect the incidence rates of these complications (27, 28). We further investigated the patients and found that those who presented post-operative electrolyte disturbances, hypothalamic syndrome, or death had more severe compression on the anterior hypothalamus than those who did not (Grade 2 on the image: 11/23 vs. 60/162). The difference between the apoplexy group and the non-apoplexy group may be partially explained by the compression.

For endocrinological aspects, apoplexy patients showed a lower LH level. It is reported that patients with pituitary adenoma apoplexy presented gonadotrophin deficiency with a percentage of 79% (29). Pituitary disorders were frequently seen in patients with CP, which was mainly caused by tumor compression (30). In our study, patients with pituitary deficiency at admission accounted for 56.89% in the non-hemorrhage group and 83.33% in the hemorrhage group. Furthermore, it was reported that in men with severe hypertension, primary hypogonadism may be a risk factor of major cardiovascular diseases (31). Decreased LH may affect the vascular tissue and provoke bleeding by the extragonadally expressed lutropin/choriogonadotropin receptor (LHR) (32, 33). Our results suggested that LH may serve as indicators together for the pituitary damage and the hemorrhage risk. Interestingly, we only found a significant difference of the LH level in male patients. This may result from the fluctuation of sexual hormones along the menstrual period in female patients, which may also explain for the difference of the T level in male patients instead of the E_2_ level and the P level in female patients.

We analyzed the correlations between the hormones and the tumor and hematoma volumes in patients with hemorrhage. A lower level of PRL was correlated with a larger volume of the tumor. Cortisol 8AM level was indicated to be positively correlated with hematoma volume. The increase of serum PRL level can be found in patients with non-PRL-secreting tumors due to hypothalamic disorders (34). In the current study, five patients had an increased PRL level before the operation. The etiology of hyperprolactinemia was probably the reduction of dopamine from the hypothalamus, which is the dominant negative hypothalamic regulation on PRL secretion (35). Impairment on the stalk function appeared but within a limited range in the 18 patients, as most of them had a normal or slightly decreased level of TSH. The negative correlation between tumor volume and PRL may attribute to hypothalamic compression which disturbed the tonic release of dopamine (30). When the 18 patients with CP apoplexy were divided into two halves according to their PRL levels, patients with lower PRL levels showed a higher incidence rate of suprasellar positioning (7/9 vs. 4/9) and grade 1 or 2 hypothalamic involvement (8/9 vs. 7/9). For cortisol, we found decreased serum levels in 13 patients and normal levels in 5 patients. According to previous reports, both increased and decreased serum levels of adrenocorticotropic hormone (ACTH), the primary regulator of cortisol releasing, could be found in CP patients (36, 37). Similar to the method for PRL analysis, we found that patients with higher cortisol levels showed a higher incidence rate of suprasellar positioning (7/9 vs. 4/9) and grade 1 or 2 hypothalamic involvement (9/9 vs. 6/9). However, because the serum levels of ACTH were missing in the recording of early patients, attribution to the disturbance of the release of CRH and ACTH is not convincing for the relation between cortisol and the volume of the hematoma. An increase of cortisol level in patients with hemorrhage may also result from stress reaction. The practical meaning and a better explanation require further research.

Our study suggests that patients with CP apoplexy could present the higher incidence rates of drowsiness, acute onset within 3 days, high PT, lower LH, and T in male patients, and pituitary deficiency than non-hemorrhagic patients. Pre-operative chiasm compression appeared more often in patients with hemorrhage. In the terms of post-operative complications, electrolyte disturbances, hypothalamic syndrome, and death were more frequent in CP apoplexy patients. Proper treatment should be applied to these patients, such as intraoperative processing of the blood clots, continuous monitoring, and individualized electrolyte supplementation after the surgery. The steady control of ICP and prompt handling of the hypothalamic syndrome with replacement therapy are also important to the prognosis of patients with CP apoplexy. To the best of our knowledge, this is the first article that compares the clinical characteristics of CP between non-hemorrhage and hemorrhage patients with the currently largest cohort of this syndrome.

We acknowledge the limitation of the current study. This is a case-control study based on retrospectively collected data accompanied by selection bias from a single clinical center. Besides, CP tumoral hemorrhage is a rare situation in clinical practice. Only 18 patients with CP apoplexy were included in the current study, which sets a limit to the cohort scale of the hemorrhage group. Routine laboratory examinations were difficult to conduct in patients with emergency admission, which introduced some missing values and case exclusion in the study.

## Conclusion

Craniopharyngioma with tumoral hemorrhage is a very rare syndrome in clinical practice. The current study presented clinical features of CP apoplexy from the aspects of clinical characteristics, radiography, surgical treatment, and post-operative complications. Patients with CP apoplexy could benefit from proper processing of peritumoral hemorrhage and post-operative monitoring of the electrolyte.

## Data Availability Statement

The raw data supporting the conclusions of this article will be made available by the authors, without undue reservation.

## Ethics Statement

The studies involving human participants were reviewed and approved by Institutional Review Board of Tongji Hospital. Written informed consent to participate in this study was provided by the participants' legal guardian/next of kin.

## Author Contributions

YC: data curation, methodology, visualization, and writing—original draft. FH: methodology, resources, supervision, and writing—review and editing. JW and KH: investigation and resources. WL, YT, KZ, and QX: investigation. TL: conceptualization and project administration. KS: conceptualization, funding acquisition, project administration, and writing—review and editing. All authors contributed to the article and approved the submitted version.

## Funding

This study was supported by the Natural Science Foundation of Hubei Province, China (2020CFB657).

## Conflict of Interest

The authors declare that the research was conducted in the absence of any commercial or financial relationships that could be construed as a potential conflict of interest.

## Publisher's Note

All claims expressed in this article are solely those of the authors and do not necessarily represent those of their affiliated organizations, or those of the publisher, the editors and the reviewers. Any product that may be evaluated in this article, or claim that may be made by its manufacturer, is not guaranteed or endorsed by the publisher.
